# Response to M. Zhang

**DOI:** 10.3389/ijph.2024.1608216

**Published:** 2025-01-15

**Authors:** Valentina A. Andreeva, Stéphanie Chambaron, Cécilia Samieri, Léopold K. Fezeu

**Affiliations:** ^1^ Nutritional Epidemiology Research Unit, Sorbonne Paris Nord University, INSERM/INRAE/CNAM, Epidemiology and Statistics Research Center (CRESS), Bobigny, France; ^2^ UMR6265 Centre des Sciences du Goût et de l’Alimentation (CSGA), CNRS/INRAE/Agro Institute, University of Bourgogne, Dijon, France; ^3^ UMR1219, Université de Bordeaux, Bordeaux, France; ^4^ INSERM, Bordeaux Population Health, Bordeaux, France

**Keywords:** anxiety, insomnia, eating disorders, general population, mental multimorbidity

Dear Editors,

We are writing with regard to the letter by Mengqin Zhang [1], published in reference to our study: “Mental multimorbidity among general-population adults: sex-specific sociodemographic profiles of anxiety, insomnia, and eating disorders” [2]. We thank the author for the interest in our work which pertains to a descriptive analysis of the prevalence and sociodemographic profiles of mental multimorbidity.

As we had noted in the text, and as observed by M. Zhang, a limitation common to epidemiological research is the reliance on self-reported data provided by volunteers. In addition, we had acknowledged the inability of the SCOFF questionnaire to distinguish among eating disorder (ED) types [3]. We agree with M. Zhang that this limitation precludes a granular understanding the comorbidity of different EDs with anxiety and insomnia. Nonetheless, we wish to point out that in our sample, EDs were the least prevalent of the three mental health conditions, especially among men. Splitting EDs into different types (i.e., restrictive/anorexic, bulimic, etc.) would have jeopardized the study due to the very small number of individuals in the various pure and comorbid ED categories. Thus, despite its limitations, the SCOFF allowed us to carry out our analyses with sufficient statistical power. We concur with M. Zhang that future studies, using more sensitive ED tools and possibly subtype-specific scales, could help characterize ED types and their distinct comorbidity patterns.

Next, we assessed trait anxiety by modelling STAI-T in sex-specific quartiles, fully understanding that such an approach was sub-optimal as the instrument was initially conceptualized on a continuous scale [4]. While our approach aligns with epidemiological practices [5], we recognize the potential for measurement bias and suggest conducting sensitivity analyses in subsequent research.

Whereas shedding light on the chronology of the three mental health conditions was beyond the scope of our study, we had acknowledged the inability to infer causality and had pointed out the availability of evidence of complex bidirectional and possibly mediated associations among anxiety, insomnia, and ED [6, 7]. Furthermore, M. Zhang has rightfully observed that the temporal ordering of mental disorders could impact treatment outcomes and prognosis. Thus, we fully agree that prospective research in this domain (possibly integrating objective mental health assessments and biomarker data) is warranted, as are moderation and mediation analyses whose findings could help guide targeted public health interventions.

Finally, we appreciate M. Zhang’s observation that our study makes a notable contribution to the understanding of mental health multimorbidity. The results could serve as impetus for further epidemiological and mechanistic research aimed at elucidating the underlying mechanisms of the complex relationships among mental disorders.
